# Modeling Health and Economic Outcomes of Providing Stable Housing to Homeless Adults With OUD

**DOI:** 10.1001/jamanetworkopen.2025.17103

**Published:** 2025-06-27

**Authors:** Isabelle J. Rao, Margaret L. Brandeau

**Affiliations:** 1 Department of Mechanical and Industrial Engineering, University of Toronto, Toronto, Ontario, Canada; 2 Department of Management Science and Engineering, Stanford University, Stanford, California

## Abstract

**Importance:**

The number of people experiencing homelessness (PEH) in the US has increased substantially in recent years. The leading cause of death among PEH is drug overdose, with opioids accounting for the majority of such deaths.

**Objective:**

To assess the costs and health outcomes of providing stable housing to PEH who have opioid use disorder (OUD).

**Design, Setting, and Participants:**

This economic evaluation conducted a model-based cost-effectiveness analysis of PEH with OUD in the US.

**Exposure:**

Provision of stable housing, with no requirement to enter OUD treatment.

**Main Outcomes and Measures:**

Primary outcomes were overdoses and deaths over 5 years, lifetime per-person discounted quality-adjusted life-years (QALYs) and costs, and incremental cost-effectiveness ratios (ICERs) compared with the status quo (no housing provision).

**Results:**

In a model of 1000 PEH (700 male; mean [SD] age, 46.4 [14.0]; 300 female; mean [SD] age, 46.5 [14.3]), under the status quo, 191 (95% CI, 152-237) deaths occurred over 5 years (58 [95% CI, 44-78] from overdose and 133 [95% CI, 101-167] from other causes). With the housing intervention, 140 (95% CI, 114-185) deaths occurred (53 [95% CI, 39-76] from overdose and 87 [95% CI, 73-110] from other causes). The housing intervention was associated with a gain of 3.59 (95% CI, 3.13-3.98) lifetime QALYs per person at an incremental cost of $26 800 (95% CI, $21 200-$32 300) per QALY gained compared with the status quo. Over extensive sensitivity analyses, the ICER remained less than $90 000 per QALY gained.

**Conclusions and Relevance:**

This economic evaluation found that investing in stable housing for this marginalized population, even with no requirement to enter OUD treatment, was associated with cost-effectiveness, fewer deaths, and improved health outcomes. Efforts are urgently needed to improve the health of PEH with OUD; it is essential to understand the outcomes and cost-effectiveness of housing provision for this marginalized population because housing status is a key social determinant of health.

## Key Points

**Question:**

What are the costs and health outcomes of providing stable housing to people experiencing homelessness (PEH) who have opioid use disorder (OUD)?

**Findings:**

In this economic evaluation using a dynamic model to assess the cost-effectiveness of providing stable housing to 1000 PEH with OUD with no requirement to enter OUD treatment, stable housing was associated with a reduction in 5-year deaths and an increase in lifetime quality-adjusted life-years per person, at an incremental cost of $26 800 per quality-adjusted life-year compared with the status quo.

**Meaning:**

These findings suggest that investing in stable housing for this marginalized population, even without requiring OUD treatment, is both life-saving and cost-effective.

## Teaser

This economic evaluation analyzes the costs and health outcomes of providing stable housing to people experiencing homelessness who have opioid use disorder (OUD).

## Introduction

The number of people experiencing homelessness (PEH) in the US has increased substantially in recent years. A 2023 point-in-time count estimated that more than 650 000 people in the US were experiencing homelessness, an increase of 12% since 2022 and the highest number since the point-in-time count began in 2007.^1^ Between 2015 and 2023, the share of PEH who were unsheltered (ie, sleeping in places not meant for human habitation such as parks and sidewalks) rose from 31% to 40%.^1,2^ In California, which has the largest unhoused population of any state with an estimated 160 000 PEH, approximately 70% of such individuals are unsheltered.^3^

PEH have high rates of premature mortality due to factors such as substance use, communicable diseases, unintentional injuries, violence, and premature aging.^4,5^ Homelessness, particularly unsheltered homelessness, is associated with a high prevalence of substance use disorders and mental health disorders, as well as disconnection from health care services.^2^ Homelessness has been found to worsen existing substance use disorders and to significantly increase the likelihood that an individual will develop substance use disorder.^2^ Moreover, individuals with substance use disorder are at increased risk of becoming homeless.^6^

PEH have been particularly affected by the US opioid epidemic; overdose is the leading cause of death among PEH, and opioids account for the majority of such overdose deaths.^7,8,9^ In recent years, the increasing prevalence of fentanyl in the drug supply has led to sharp increases in overdose deaths.^10,11^ In addition to accidental overdose, substance use disorders increase the risk of contracting communicable diseases such as HIV and hepatitis C virus.^5^

Receipt of treatment for opioid use disorder (OUD) is low in the US,^12^ and a recent national survey found that PEH with OUD were less likely than other adults with OUD to enter outpatient treatment or receive medication for OUD.^13^ Barriers to entry include personal barriers such as competing priorities, trauma, and medical comorbidities; practical barriers such as transportation and medication security; and structural barriers such as stigma, mistrust of medical institutions, and lack of health insurance coverage.^14,15^

Many efforts have been undertaken to provide housing for PEH, including the provision of emergency shelters and transitional and permanent housing, accompanied by subsidies and a varying range of supportive services. In the treatment first approach, individuals must be treated for underlying addiction and mental health issues and/or refrain from substance use to enter and stay in permanent housing. In the housing-first approach, PEH are provided housing without a requirement for sobriety or receipt of treatment.^16^ Housing first is now endorsed by the US Interagency Council on Homelessness,^17^ and until recently California has required all tax dollars used for housing to endorse a housing first model. Elements of the housing-first approach include immediate access to housing and supportive services, a harm reduction approach with no requirement for sobriety or participation in treatment, intensive case management, and access to and coordination with OUD treatment and educational, employment, mental health, and other social services.^18^

In this study, we developed and applied a dynamic model to estimate the potential costs and health outcomes of a housing-first intervention that provides stable housing to PEH with OUD. We calculated lifetime costs and quality-adjusted life-years (QALYs), and the number of overdose deaths and total deaths over a 5-year time horizon. We compared these outcomes with those of PEH with OUD who were not provided with stable housing.

## Methods

### Overview

**Figure 1.**
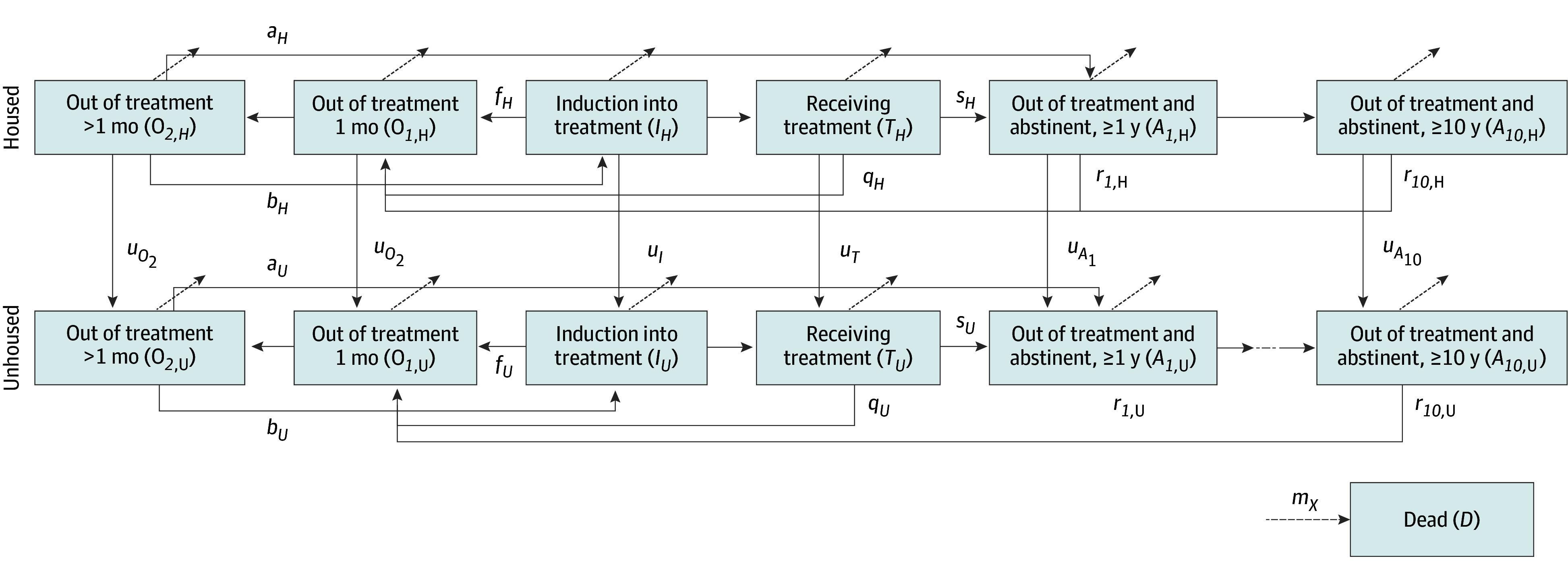
Model Schematic In this model schematic, the state in each box is abbreviated in parentheses. A subscript *H* indicates housed, and a subscript *U* indicates unhoused. Variable *a* indicates the rate at which untreated individuals become abstinent; *b*, the rate at which individuals begin treatment; *f,* the failure rate from induction into treatment state; *m*, the mortality rate; *q*, the rate at which individuals quit treatment; *r*, the relapse rate; and *s*, the rate at which individuals successfully leave treatment and become abstinent.

This economic evaluation was determined exempt by the Stanford University institutional review board and followed the Consolidated Health Economic Evaluation Reporting Standards (CHEERS) reporting guideline. We developed a continuous time compartmental model to simulate housing status, opioid use, and OUD treatment (Figure 1). We modeled a representative cohort of PEH with OUD in the US, with sex and age distribution as reflected in recent surveys.^19,20,21^ We simulated the model over the lifetime of the cohort and calculated the number of overdoses and deaths that occur over 5 years as well as lifetime per-person discounted health care costs and QALYs. Parameter values and sources are shown in Table 1. We took a limited societal perspective that includes housing cost plus all health care costs, and followed standard guidelines for conducting cost-effectiveness analysis, with costs and health outcomes discounted at 3% annually.^22^ Full details of the model, parameter estimation, and model instantiation are provided in the eMethods in Supplement 1.

**Table 1.** Base-Case Parameter Values and Sources

Parameter	Value (range)	Source
Demographics		
Sex, No. (%)		
Male	700 (70)	Kushel et al,^19^ 2023; Substance Abuse and Mental Health Services Administration,^20^ 2024; US Census Bureau,^21^
Female	300 (30)	Kushel et al,^19^ 2023; Substance Abuse and Mental Health Services Administration,^20^ 2024; US Census Bureau,^21^
Male age, mean (SD), y	46.4 (14.0)	Kushel et al,^19^ 2023; Substance Abuse and Mental Health Services Administration,^20^ 2024; US Census Bureau,^21^
Female age, mean (SD), y	46.5 (14.3)	Kushel et al,^19^ 2023; Substance Abuse and Mental Health Services Administration,^20^ 2024; US Census Bureau,^21^
Death and overdose, annual rates per person^a^		
Background mortality	Centers for Disease Control and Prevention life tables	Arias et al,^54^ 2023
Nonoverdose excess mortality due to OUD, out of treatment		
Housed	0.0098 (0.0074-0.0125)	Ma et al,^48^ 2019
Unhoused	0.0032 (0.0024-0.0041)	Ma et al,^48^ 2019; Morrison et al, ^53^ 2009
Excess mortality due to homelessness		
Out of treatment	0.0128 (0.0065-0.0203)	Morrison et al, ^53^ 2009
In treatment	0.0055 (0.0028-0.0084)	Morrison et al, ^53^ 2009
Multiplier for increased all-cause mortality when inducted onto methadone	14.0 (1.07-62.45)	Ma et al,^48^ 2019
Overdose, housed		
Out of treatment	0.1331 (0.0652-0.2605)	Fairley et al,^23^ 2019; Quian et al, ^24^ 2021; Kelty et al,^49^ 2019; Lim et al,^50^ 2022
In treatment	0.0567 (0.0283-0.1098)	Fairley et al,^23^ 2019; Quian et al, ^24^ 2021; Kelty et al,^49^ 2019; Lim et al,^50^ 2022
Overdose, unhoused		
Out of treatment	0.1368 (0.0665-0.2700)	Fairley et al,^23^ 2019; Quian et al, ^24^ 2021; Kelty et al,^49^ 2019; Lim et al,^50^ 2022
In treatment	0.0583 (0.0288-0.1137)	Fairley et al,^23^ 2019; Quian et al, ^24^ 2021; Kelty et al,^49^ 2019; Lim et al,^50^ 2022
Overdose survival probability per overdose		
Housed	0.880 (0.794-0.943)	Quian et al, ^24^ 2021; Lim et al,^50^ 2022; Booth et al,^51^ 2024; Coffin et al,^52^ 2013
Unhoused	0.883 (0.798-0.945)	Quian et al, ^24^ 2021; Lim et al,^50^ 2022; Booth et al,^51^ 2024; Coffin et al,^52^ 2013
OUD and treatment transitions, annual rates per person^a^		
Entry into treatment (from out of treatment >1 mo)		
Housed	0.426 (0.367-0.489)	Krebs et al,^34^ 2017
Unhoused	0.213 (0.184-0.245)	Krebs et al,^34^ 2017; Corsi et al,^38^ 2007; Deck et al,^39^ 2004; Rivers et al,^40^ 2006; Dunn et al,^41^ 2019; Krawczyk et al,^42^ 2020; Royse et al,^43^ 2000; Simon et al,^44^ 2017
Treatment discontinuation, methadone (return to untreated OUD)		
Housed	1.051 (0.578-1.740)	Hser et al,^35^ 2014; Neumann et al,^36^ 2013; Potter et al,^37^ 2013
Unhoused	1.431 (0.783-2.378)	Hser et al,^35^ 2014; Neumann et al,^36^ 2013; Potter et al,^37^ 2013; Fine et al,^45^ 2021
Treatment discontinuation, buprenorphine (return to untreated OUD)		
Housed	1.608 (1.004-2.420)	Hser et al,^35^ 2014; Neumann et al,^36^ 2013; Potter et al,^37^ 2013
Unhoused	2.189 (1.361-3.290)	Hser et al,^35^ 2014; Neumann et al,^36^ 2013; Potter et al,^37^ 2013; Fine et al,^45^ 2021
Becoming abstinent and leaving treatment		
Housed	0.316 (0.296-0.337)	Krebs et al,^34^ 2017
Unhoused	0.029 (0.028-0.031)	Krebs et al,^34^ 2017; Fine et al,^45^ 2021
Becoming abstinent from out of treatment		
Housed	0.0791 (0.0040-0.1549)	Fairley et al,^23^ 2019
Unhoused	0.0074 (0.0040-0.0144)	Fairley et al,^23^ 2019; Fine et al,^45^ 2021
Relapse from abstinence <1 y		
Housed	0.344 (0.300-0.390)	Krebs et al,^34^ 2017
Unhoused	0.462 (0.404-0.524)	Krebs et al,^34^ 2017; Goldman-Hasbun J, et al,^46^ 2019; Kertesz et al,^47^ 2003
Relapse from abstinence >1 y		
Housed	0.023 (0.0047-0.0419)	Krebs et al,^34^ 2017
Unhoused	0.031 (0.0063-0.0564)	Krebs et al,^34^ 2017; Goldman-Hasbun J, et al,^46^ 2019; Kertesz et al,^47^ 2003
Housing transitions		
Becoming unhoused, annual rate per person^a^	0.0820 (0.0739-0.0903)	Pfefferle et al,^18^ 2019; Montgomery et al,^26^ 2013; Stefancic et al,^27^ 2013; Pearson et al,^28^ 2009; Collins et al,^29^ 2013
Costs, $^b^		
Annual background health care costs		
Baseline, male aged 30 y	2764	Liu et al,^55^ 2012; Meara et al,^56^ 2004
Excess health care cost for OUD		
Out of treatment	8827 (7966-9731)	Baser et al,^58^ 2014
Receiving treatment	7052 (3675-10 658)	Baser et al,^57^ 2011
Excess health care cost for homelessness	13 823 (12 475-15 257)	Jacob et al,^31^ 2022
Other costs^b^		
Annual cost of housing	20 900	Jacob et al,^31^ 2022; Larimer et al,^32^ 2009
Annual treatment costs for methadone	8584 (7747-9464)	Department of Defense,^33^ 2016
Annual treatment costs for buprenorphine	7836 (7077-8629)	Department of Defense,^33^ 2016
Naloxone cost, per initial provision or refill	87 (79-96)	Fairley et al,^23^ 2019
Health care cost per overdose		
Housed	3084 (1321-6152)	Booth et al,^51^ 2024; Coffin et al,^52^ 2013
Unhoused	3029 (1297-6043)	Booth et al,^51^ 2024; Coffin et al,^52^ 2013
Quality of life multipliers		
Out of treatment (first month)		
Housed	0.668 (0.655-0.680)	Fairley et al,^23^ 2019; Krebs et al,^61^ 2018; Nosyk et al,^62^ 2015
Unhoused	0.290 (0.284-0.295)	Fairley et al,^23^ 2019; Krebs et al,^61^ 2018; Nosyk et al,^62^ 2015; Rajan et al,^63^ 2021
Out of treatment (>1 mo)		
Housed	0.668 (0.655-0.680)	Fairley et al,^23^ 2019; Krebs et al,^61^ 2018; Nosyk et al,^62^ 2015
Unhoused	0.290 (0.284-0.295)	Fairley et al,^23^ 2019; Krebs et al,^61^ 2018; Nosyk et al,^62^ 2015; Rajan et al,^63^ 2021
Induction into treatment		
Housed	0.721 (0.700-0.742)	Fairley et al,^23^ 2019; Krebs et al,^61^ 2018; Nosyk et al,^62^ 2015
Unhoused	0.313 (0.304-0.322)	Fairley et al,^23^ 2019; Krebs et al,^61^ 2018; Nosyk et al,^62^ 2015; Rajan et al,^63^ 2021
Receiving treatment		
Housed	0.721 (0.700-0.742)	Fairley et al,^23^ 2019; Krebs et al,^61^ 2018; Nosyk et al,^62^ 2015
Unhoused	0.313 (0.304-0.322)	Fairley et al,^23^ 2019; Krebs et al,^61^ 2018; Nosyk et al,^62^ 2015; Rajan et al,^63^ 2021
Abstinence (first year)		
Housed	0.747 (0.728-0.766)	Fairley et al,^23^ 2019; Fryback et al,^60^ 2007; Krebs et al,^61^ 2018
Unhoused	0.324 (0.316-0.333)	Fairley et al,^23^ 2019; Fryback et al,^60^ 2007; Krebs et al,^61^ 2018; Rajan et al,^63^ 2021
Abstinence (≥10 y)		
Housed	0.983 (0.970-0.997)	Fairley et al,^23^ 2019; Fryback et al,^60^ 2007; Krebs et al,^61^ 2018
Unhoused	0.427 (0.421-0.433)	Fairley et al,^23^ 2019; Fryback et al,^60^ 2007; Krebs et al,^61^ 2018; Rajan et al,^63^ 2021

Abbreviation: OUD, opioid use disorder.

^a^ Annual rate per person refers to the expected number of occurrences of an event per year and per person.

^b^ All costs are expressed in 2024 USD.

### Statistical Analysis

#### Model Dynamics

We considered a cohort of PEH with OUD who were currently not receiving OUD treatment. In the status quo, all individuals start as unhoused and not receiving treatment for more than 1 month. We considered a housing intervention where all individuals in the cohort were provided with stable housing and had not been receiving treatment for more than 1 month. Individuals in any housed compartment could leave housing and move to the equivalent unhoused compartment (Figure 1). We assumed that, once unhoused, an individual does not enter housing again over the simulated time horizon but may enter and leave OUD treatment. We modeled the flow of individuals into and out of OUD treatment in a manner similar to previous studies of OUD treatment.^23,24,25^ Individuals could transition between different health states (out of treatment, receiving treatment, and out of treatment and abstinent), and different housing states (housed and unhoused).

#### Housing Intervention

We considered a 1-time housing intervention where the cohort of PEH was provided with permanent supportive housing at the beginning of the simulated time horizon and compared this with the status quo where the cohort remained unhoused. We assumed a housing-first approach that does not require housed individuals to enter OUD treatment or achieve sobriety, and includes the key elements described previously (eg, intensive case management and access to OUD treatment and other services).^18^

Several studies have examined retention in housing-first programs,^18,26,27,28,29,30^ although none have focused specifically on PEH with OUD. Based on these studies, we estimated that the average annual rate of becoming unhoused among individuals provided with housing would be 0.082. We estimated that the annual cost of housing would be $20 900 per person housed.^31,32^

#### OUD Treatment

We assumed that OUD treatment consists of buprenorphine or methadone, along with a baseline level of drug counseling and medication management services.^33^ For housed individuals, we used OUD and treatment transition rates drawn from a previously validated model^23,24,25^ and other published studies.^34,35,36,37^ For unhoused individuals, we used hazard ratios estimated from the literature to adjust the transition rates used for housed individuals. A number of studies have shown that, compared with housed individuals, PEH have lower rates of entry into^15,38,39,40,41,42,43,44^ and retention in^45^ OUD treatment, lower treatment success^45^ (achieving abstinence), and higher rates of relapse from abstinence.^46,47^ Based on these studies, we estimated hazard ratios for PEH of 0.50 for rates of entry into treatment, 1.36 for rates of treatment discontinuation, 0.09 for rates of treatment success, and 1.35 for rates of relapse, when compared with housed individuals.

#### Deaths and Overdose

Deaths can occur from any state in the model. We distinguished background mortality, nonoverdose excess mortality due to OUD, and overdose mortality for housed and unhoused individuals.^23,24,48,49,50,51,52^ For unhoused individuals, we also considered excess mortality due to homelessness.^53^ Background mortality by age and sex was drawn from Centers for Disease Control and Prevention life tables^54^ and applied to both housed and unhoused individuals.

#### Costs and QALY Multipliers

In addition to housing cost, we measured background health care costs,^55,56^ excess health care costs associated with OUD (both in treatment^57^ and out of treatment^58^), excess health care costs associated with homelessness,^31^ costs of OUD treatment,^33^ costs of naloxone for overdoses,^23^ and health care costs per overdose.^23,52,59^ All costs are expressed in 2024 USD.

Quality of life multipliers for health states for housed individuals were estimated from several studies.^23,60,61,62^ To adjust these estimates for homelessness, a utility multiplier of 0.43, taken from a cross-sectional national survey conducted in the US,^63^ was applied.

#### Model Implementation

The model was implemented in Python version 3.8.5 (Python Software Foundation) and simulated over the lifetime of the cohort. For each simulation, model parameters were drawn stochastically from the ranges of parameter values shown in Table 1; probability distributions for the stochastic parameters are shown in eTable 1 in Supplement 1. In the base case, we performed 25 000 simulation runs for each cohort and calculated mean outcomes and 95% CIs. For each sensitivity analysis where we varied key model parameters (eg, cost of housing, rate of becoming unhoused), we performed 10 000 simulation runs.

## Results

The model included 1000 PEH (700 male; mean [SD] age, 46.4 [14.0]; 300 female; mean [SD] age, 46.5 [14.3]). Results were similar for the cases of OUD treatment with methadone (Table 2 and eTable 2 in Supplement 1) or buprenorphine (eTable 3 and eTable 4 in Supplement 1). Here we describe in detail only the cases of OUD treatment with methadone.

**Table 2.** Outcomes for Each Scenario, Assuming OUD Treatment With Methadone

Scenario	Overdoses over 5 y per 1000 individuals, mean (95% CI)				Deaths over 5 y per 1000 individuals, mean (95% CI)	Lifetime QALYs per person discounted, mean (95% CI)		Lifetime costs per person discounted, mean (95% CI), $^a^			ICER, mean (95% CI)^a^
	Fatal	Nonfatal	Total					Health care	Housing	Total	
Status quo	58 (44-78)	496 (219-1018)		554 (272-1083)	191 (152-237)		3.71 (3.26-4.14)	449 (388-507)	0 (0-0)	449 (388-507)	NA
Base-case analysis											
Housing intervention	53 (39-76)	440 (189-926)		494 (235-989)	140 (114-185)		7.30 (6.57-7.87)	396 (347-441)	148 (137-156)	545 (486-593)	26.8 (21.2-32.3)
Sensitivity analyses											
Lower treatment success for housed individuals^b^	56 (41-78)	465 (201-947)		521 (249-1008)	144 (119-184)		7.05 (6.38-7.59)	394 (343-438)	147 (136-155)	540 (482-588)	27.3 (21.6-32.7)
Housing not associated with changes in OUD treatment entry or outcomes^c^	61 (46-81)	508 (224-1056)		569 (279-1116)	151 (127-186)		6.62 (6.08-7.08)	388 (341-433)	143 (134-152)	532 (478-581)	28.5 (22.6-33.9)
Lower probability of witnessing an overdose for housed individuals^d^	54 (40-78)	438 (189-921)		492 (236-985)	141 (115-185)		7.29 (6.57-7.86)	396 (346-440)	148 (137-156)	544 (486-592)	26.6 (20.9-32.0)
Higher rate of becoming unhoused^e^	54 (40-76)	453 (199-939)		507 (248-1002)	147 (121-190)		5.97 (5.39-6.45)	417 (363-468)	94 (88-99)	511 (454-563)	27.7 (21.9-33.3)
Higher rate of becoming unhoused for nonabstinent individuals^f^	53 (39-76)	445 (192-931)		499 (238-996)	143 (117-186)		6.74 (6.06-7.32)	405 (355-452)	125 (115-133)	530 (473-580)	26.9 (21.3-32.3)
Housing not associated with changes in excess mortality due to homelessness^g^	52 (38-75)	432 (185-926)		484 (229-988)	174 (137-225)		6.84 (6.05-7.52)	372 (318-423)	139 (128-149)	511 (446-569)	19.9 (15.6-24.0)
Doubled cost of housing	53 (39-76)	441 (190-915)		494 (237-978)	139 (114-183)		7.30 (6.58-7.89)	397 (346-441)	297 (276-313)	694 (625-747)	68.3 (62.3-74.5)

Abbreviations: ICER, incremental cost-effectiveness ratio; OUD, opioid use disorder; QALY, quality-adjusted life-year.

^a^ All costs are expressed in thousands of 2024 USD; ICER was calculated as cost per QALY gained compared with the status quo.

^b^ Rate of entry into treatment and rate of becoming abstinent was 20% lower, and rate of treatment discontinuation and rate of relapse from abstinence was 20% higher.

^c^ Rates of entry into treatment and treatment outcomes for housed individuals are the same as for unhoused individuals.

^d^ Probability of witnessing an overdose for housed individuals was 80% lower than for housed individuals.

^e^ Rate of becoming unhoused was twice as high.

^f^ The rate of becoming unhoused was 50% higher for individuals out of treatment, 25% higher for those in treatment, and unchanged for abstinent individuals.

^g^ Excess mortality due to homelessness for housed individuals was the same as for unhoused individuals.

### Base-Case Analyses

**Figure 2.**
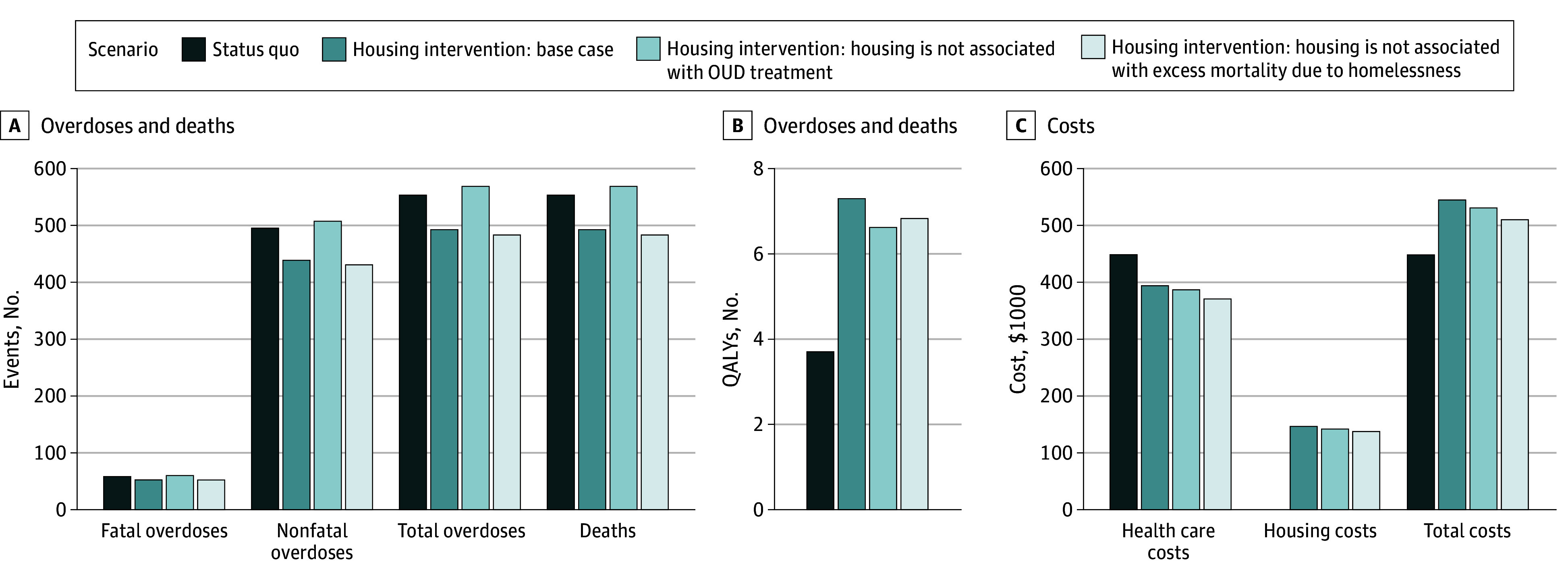
Outcomes for the Status Quo and Housing Intervention Under 3 Scenarios, Assuming Opioid Use Disorder (OUD) Treatment With Methadone QALY indicates quality-adjusted life-year.

Under the status quo without the housing intervention, 191 (95% CI, 152-237) deaths occurred in the cohort of 1000 individuals over 5 years (Table 2 and Figure 2). A total of 554 (95% CI, 272-1083) overdoses occurred, including 58 (95% CI, 44-78) fatal and 496 (95% CI, 219-1018) nonfatal. The remaining 133 (95% CI, 101-167) deaths were due to other causes. Each person experienced 3.71 (95% CI, 3.26-4.14) discounted lifetime QALYs and incurred $449 000 (95% CI, $388 000-$507 000) in discounted lifetime health care costs.

With the housing intervention, 140 (95% CI, 114-185) deaths occurred in the cohort over 5 years, 51 (95% CI, 26 to 77) fewer than with no housing intervention; additionally, 5 (95% CI, −3 to 11) fewer fatal overdoses occurred and 46 (95% CI, 24-70) fewer deaths from other causes occurred (Table 2 and Figure 2). The housing intervention was associated with an 11% reduction in total overdoses (494 [95% CI, 235-989] vs 554 [95% CI, 272-1083] overdoses), a 9% reduction in fatal overdoses (53 [95% CI, 39-76] vs 58 [95% CI, 44-78] overdoses), and a 35% reduction in deaths from other causes (87 [95% CI, 73-110] vs 133 [95% CI, 101-167] deaths) over 5 years. A total of 7.30 (95% CI, 6.57-7.87) discounted lifetime QALYs were experienced per person, a gain of 3.59 (95% CI, 3.13-3.98) QALYs compared with the status quo. Lifetime per-person costs were $545 000 (95% CI, $486 000-$593 000), comprising $396 000 (95% CI, $347 000-$441 000) in health care cost and $148 000 (95% CI, $137 000-$156 000) in housing cost, leading to a total incremental cost of $96 000 (95% CI, $72 000-$119 000) compared with the status quo. Thus, the housing intervention cost $26 800 (95% CI, $21 200-$32 300) per QALY gained.

### Sensitivity Analyses

We performed a variety of sensitivity analyses in which we varied uncertain values in a manner that could make the housing intervention appear less favorable. We present results for key sensitivity analyses here and discuss additional analyses in the eResults in Supplement 1.

#### Association of Housing With OUD Treatment

The base case assumed rates of OUD treatment entry and treatment success that reflect an average individual with OUD, regardless of whether they have been unhoused. If treatment entry and success for the newly housed individuals was lower than we estimated in the base case—in particular, if the rate of entry into treatment was decreased by 20%, the rate of treatment discontinuation was increased by 20%, the rate of becoming abstinent (in or out of treatment) was decreased by 20%, and the rate of relapse from abstinence was increased by 20%—then 144 (95% CI, 119-184) deaths occurred over 5 years (compared with 140 [95% CI, 114-185] deaths in our base-case analysis of the housing intervention and 191 [95% CI, 152-237] with no housing intervention) (Table 2). Total lifetime QALYs were 7.05 (95% CI, 6.38-7.59) per person, and total costs were $540 000 (95% CI, 482 000-$588 000). Compared with the status quo, the housing intervention cost $27 300 (95% CI, $21 600-32 700) per QALY gained.

If the provision of housing was not associated with an increased chance of entry into treatment—that is, if rates of entry into treatment and treatment outcomes for housed individuals were the same as for unhoused individuals—then 569 (95% CI, 279-1116) overdoses and 151 (95% CI, 127-186) deaths occurred over 5 years (compared with 494 [95% CI 235-989] overdoses and 140 [95% CI, 114-185] deaths in our base-case analysis and 554 [95% CI, 272-1083] overdoses and 191 [95% CI, 152-237] deaths with no housing intervention) (Table 2 and Figure 2). Overdoses increased compared with the status quo because fewer individuals died from being unhoused and thus more individuals were likely to overdose, but deaths were lower than in the status quo because fewer individuals died from being unhoused. Total lifetime QALYs were 6.62 (95% CI, 6.08-7.08) per person, and total costs were $532 000 (95% CI, $478 000-$581 000). A total of 2.91 (95% CI, 2.65-3.15) lifetime QALYs were gained compared with the status quo. These QALY gains accrued from the lower death rate and higher QALY multiplier of housed vs unhoused individuals. Compared with the status quo, the housing intervention cost $28 500 (95% CI, $22 600-$33 900) per QALY gained.

#### Chance of Overdose Being Witnessed

If the probability of witnessing an overdose for housed individuals was 80% lower than in the base case, outcomes were essentially unchanged. With this probability, 141 (95% CI, 115-185) deaths occurred, and lifetime per person QALYs and costs were 7.29 (95% CI, 6.57-7.86) and $544 000 (95% CI, $486 000-$592 000), respectively, leading to an incremental cost of $26 600 (95% CI, $20 900-$32 000) per QALY gained compared with the status quo (Table 2).

#### Rate of Becoming Unhoused

If the rate of becoming unhoused was twice as high as in the base case, 147 (95% CI, 121-190) deaths occurred, and per person QALYs and costs decreased to 5.97 (95% CI, 5.39-6.45) and $511 000 (95% CI, $454 000-$563 000), respectively (Table 2). In this case, the housing intervention cost $27 700 (95% CI, $21 900-$33 300) per QALY gained compared with the status quo.

If the rate of becoming unhoused was 50% higher for individuals out of treatment, 25% higher for those in treatment, and unchanged for abstinent individuals, 143 (95% CI, 117-186) deaths occurred, and per person QALYs and costs decreased to 6.74 (95% CI, 6.06-7.32) and $530 000 (95% CI, $473 000-580,000), respectively, resulting in an incremental cost of $26 900 (95% CI, $21 300-$32 300) per QALY gained compared with the status quo (Table 2).

#### Excess Mortality Due to Homelessness

The base case assumed mortality rates for housed individuals that reflect an average housed individual, regardless of whether they have been unhoused. To account for the potential long-term health outcomes of previous homelessness, we assumed in sensitivity analysis that excess mortality due to homelessness for housed individuals was the same as for unhoused individuals. In this case, 174 (95% CI, 137-225) deaths occurred, and per person QALYs and costs decreased to 6.84 (95% CI, 6.05-7.52) and $511 000 (95% CI, $446 000-$569 000), respectively, leading to an incremental cost of $19 900 (95% CI, $15 600-$24 000) per QALY gained compared with the status quo (Table 2 and Figure 2).

For the previous sensitivity analyses (smaller-magnitude association of housing with treatment, lower chance of overdose being witnessed, higher rate of becoming unhoused, no association of housing with reduced excess mortality due to homelessness), more deaths occurred with the housing intervention compared with the base case, but total cost was lower. The higher cost in the base case was due to the ongoing need for housing and related services for housed individuals who survived due to the improved outcomes from the intervention.

#### Higher QALYs for PEH

The baseline quality-of-life adjustment associated with being unhoused of 0.43 was drawn from a US national study.^63^ In sensitivity analysis, we increased this value to 0.60. In this case, net present lifetime QALYs increased to 5.13 (95% CI, 4.49-5.72) in the status quo vs 3.71 (95% CI, 3.26-4.14) in the base-case status quo and to 8.13 (95% CI, 7.28-8.83) with the housing intervention vs 7.30 (95% CI, 6.57-7.87) in the base case, leading to an incremental cost of $32 000 (95% CI, $25 500-$38 100) per QALY gained for the housing intervention (eTable 2 in Supplement 1). QALY gains from becoming housed were lower than in the base case (3.00; 95% CI, 2.52-3.45 vs 3.59; 95% CI, 3.13-3.98) but the housing intervention was still cost-effective because significant numbers of deaths were prevented.

#### Housing Cost and Excess Health Care Cost When Unhoused

Results were particularly sensitive to costs of housing and health care for PEH. If the cost of housing was twice as high as in the base case (thus, $41 800 per person annually), health outcomes were unchanged but total cost increased to $694 000 (95% CI, $625 000-$747 000) (Table 2), and the housing intervention cost $68 300 (95% CI, $62 300-$74 500) per QALY gained compared with the status quo. If the annual housing cost was less than $83 000, approximately 4 times as high as we estimated in the base case, the housing intervention still cost less than $150 000 per QALY gained compared with the status quo.

**Figure 3.**
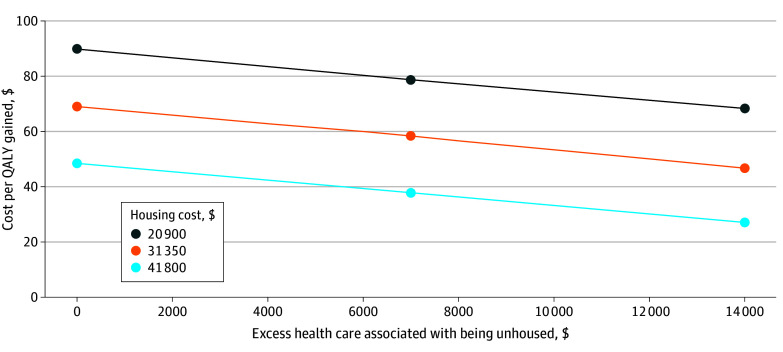
Two-Way Sensitivity Analysis: Cost of Housing and Excess Health Care Cost Associated With Being Unhoused QALY indicates quality-adjusted life-year.

We performed 2-way sensitivity analyses on housing cost and excess health care cost associated with being unhoused (eTable 2 in Supplement 2 and Figure 3). We considered the conservative cases of 50% lower health care cost associated with being unhoused or no excess health care cost associated with being unhoused (vs $13 823 annual cost in the base case), along with the cases of baseline housing cost ($20 900 per year), 50% higher housing cost, and doubled housing cost. In all cases, the housing intervention cost less than $90 000 per QALY gained compared with the status quo.

## Discussion

Our economic evaluation found that a 1-time housing intervention for PEH with OUD, with no requirement for OUD treatment or sobriety, was associated with a reduction in overdoses and deaths and an increase in QALYs—on the order of 3 to 3.5 lifetime net present QALYs per person—at a cost less than $90 000 per QALY gained, even under pessimistic assumptions. The QALY gains stemmed from the significantly higher quality of life for housed vs unhoused individuals, a lower baseline risk of dying, and a potentially higher chance of entering and remaining in OUD treatment. Our dynamic model enabled us to capture the effect sizes of these interacting factors.

Budgetary impact is also important to consider. We estimated an annual housing cost of $20 900 per person housed, but not all housed individuals will remain housed. Accounting for attrition from housing back to homelessness, we estimated a first-year housing cost of $17 948 per person housed and annual health care costs per person housed of $17 142, a savings of $11 446 from the status quo health care cost of $28 588 per person. Additionally, savings in criminal justice costs would accrue. PEH frequently have high criminal justice system costs (primarily for jail stays)—on the order of $5000 to $15 000 or more per person annually^32,64,65,66,67,68^—costs that would be reduced by housing provision.

For a city such as San Jose, California, the estimated annual cost to house the city’s estimated 6340 PEH^69^ would thus be $113.8 million, while an estimated $72.6 million in health care savings would be accrued. In addition, if all PEH who became housed incurred $5000 less in criminal justice system cost annually, housing the 6340 PEH would lead to $31.7 million in annual criminal justice system savings.

Our analysis assumes that housing for PEH is available. Construction of new housing would be a substantial additional expense. The cost of affordable housing varies from $150 000 to $1 million per unit nationwide.^68,70^ The city of San Jose, California, currently spends $225 000 per unit of affordable housing built.^69^ To house all 6340 PEH, the estimated construction cost would be $1.43 billion.

### Limitations

This study has several limitations. Available data on many factors associated with being unhoused (eg, rate of OUD treatment entry) and on the outcomes of housing for PEH with OUD (eg, changes in mortality when an unhoused individual becomes housed) are sparse. We developed parameter value estimates based on a comprehensive literature review, and we performed extensive sensitivity analyses, but further data would enable us to refine our cost-effectiveness estimates.

Our cost-effectiveness analysis did not include criminal justice savings, which would make the housing intervention appear more favorable than we had estimated. We assumed a constant housing cost. If potential decreases over time in service-related housing costs for individuals who remain in housing were considered, the housing intervention would appear more favorable. We did not model the association of housing provision with prevention, detection, and treatment of HIV and hepatitis C virus, common co-occurring conditions among PEH with OUD.^5^ Inclusion of these conditions would increase the estimated health benefits of housing provision.

We have modeled a representative 1-time housing intervention for a cohort of PEH with OUD. However, housing programs vary widely in terms of clients served, services offered, and housing type (eg, single-room occupancy in scattered locations vs housing in a single site), as well as costs and outcomes. To reflect this variability, we performed extensive sensitivity analyses, but specific programs may differ from those we have considered. Additionally, we used a relatively simple compartmental model, which is useful for calculating population averages, but does not take into account heterogeneity in housing status (eg, temporarily unhoused vs chronically unhoused or unhoused vs unsheltered), drug use status (eg, injection vs noninjection drug use), co-occurring conditions (eg, mental illness or additional substance use such as alcohol or methamphetamines), or other demographic factors (eg, age, sex, and race). A more detailed microsimulation could reflect this heterogeneity, enabling estimation of outcomes for specific populations of PEH with OUD, but would require substantial additional data.

We have evaluated a housing-first intervention that places people into permanent supportive housing with no preconditions. However, the housing-first approach may not be effective in reducing substance use for all unhoused populations.^71,72^ In the current era of widespread fentanyl, continued substance use can have substantial consequences; for example, a recent analysis^73^ found that an average of 3 people die of drug overdoses every week in permanent supportive housing in San Francisco. Some policymakers have thus developed renewed interest in a treatment first approach, where individuals must be treated for underlying addiction and mental health issues and/or refrain from substance use to enter and stay in permanent housing. A bill passed in California in 2024 allows up to 25% of taxpayer funds for housing to be spent on drug-free recovery housing, the same proportion recommended in guidance from the US Department of Housing and Urban Development.^74^ A model assessing costs and health outcomes of a treatment first intervention would need to incorporate the sequential steps in the recovery and housing process, possibly using a microsimulation.

## Conclusions

Efforts are urgently needed to improve the health of PEH with OUD. Our economic evaluation suggests that investing in stable housing for this marginalized population, even with no requirement to enter OUD treatment, not only saves lives and improves health outcomes but is cost-effective. The development of housing options for PEH, including PEH with OUD, should be an urgent national priority.

## Notes

Supplement 1.
eMethods.
**eTable 1.** Parameter Distributions **eTable 2.** Sensitivity Analysis: Outcomes Assuming OUD Treatment With Methadone **eTable 3.** Outcomes for Each Scenario, Assuming OUD Treatment With Buprenorphine **eTable 4.** Sensitivity Analysis: Outcomes Assuming OUD Treatment With Buprenorphine
eResults.

eReferences.

Supplement 2.
Data Sharing Statement
