# Supplementary material for: Modeling Health and Economic Outcomes of Providing Stable Housing to Homeless Adults With OUD
Source: JAMA Netw Open. 2025 Jun 27;8(6):e2517103. doi: 10.1001/jamanetworkopen.2025.17103 (PMC12205399; doi:10.1001/jamanetworkopen.2025.17103)
Supplement: Supplement 1. — eMethods. eTable 1. Parameter Distributions eTable 2. Sensitivity Analysis: Outcomes Assuming OUD Treatment With Methadone eTable 3. Outcomes for Each Scenario, Assuming OUD Treatment With Buprenorphine eTable 4. Sensitivity Analysis: Outcomes Assuming OUD Treatment With Buprenorphine eResults. eReferences. [file jamanetwopen-e2517103-s001.pdf]

Supplemental Online Content

Rao IJ, Brandeau ML. Modeling health and economic outcomes of providing stable housing to homeless adults with OUD. *JAMA Netw. Open*. 2025;8(6):e2517103.
doi:10.1001/jamanetworkopen.2025.17103

eMethods.
eTable 1. Parameter Distributions
eTable 2. Sensitivity Analysis: Outcomes Assuming OUD Treatment With Methadone
eTable 3. Outcomes for Each Scenario, Assuming OUD Treatment With Buprenorphine
eTable 4. Sensitivity Analysis: Outcomes Assuming OUD Treatment With Buprenorphine
eResults.
eReferences.

This supplemental material has been provided by the authors to give readers additional information about their work.

© 2025 Rao IJ et al. *JAMA Network Open*.

eMETHODS

Model Equations

Our model is a continuous time dynamic compartmental model. We define the compartments using the following notation: O denotes out of treatment, I denotes induction into treatment, T denotes treatment, A denotes abstinence (not on treatment), and D represents death. We differentiate time out of treatment and time in abstinence using subscripts. For example, O_1 denotes individuals who have been out of treatment for less than a month, and A_1 denotes individuals who have been abstinent for less than 1 year. We further distinguish individuals based on their housing status. Subscripts H and U denote housed and unhoused individuals, respectively.

O	Individuals out of treatment
I	Individuals inducted into treatment (first month of treatment)
T	Individuals in treatment
A	Abstinent individuals
D	Dead individuals
u_C	Transition rate from housed compartment C to equivalent unhoused compartment: $C_H \rightarrow C_U$, with $C \in \{O_1, O_2, I, T, A_1, \cdots, A_{10}\}$
b_X	Rate at which individuals begin treatment: $O_{2,X} \rightarrow I_X$, with $X \in \{U, H\}$
a_X	Rate at which untreated individuals become abstinent: $O_{2,X} \rightarrow A_{1,X}$, with $X \in \{U, H\}$
m_C	Mortality rate from compartment C
$r_{k,X}$	Relapse rate: $A_{k,X} \rightarrow O_{1,X}$, with $X \in \{U, H\}$ and $k \in \{1, \ldots, 10\}$
q_X	Rate at which individuals quit treatment: $T_X \rightarrow O_{1,X}$, with $X \in \{U, H\}$
f_X	Failure rate from induction into treatment state: $I_X \rightarrow O_{1,X}$, with $X \in \{U, H\}$
s_X	Rate at which individuals successfully leave treatment and become abstinent: $T_X \rightarrow A_{1,X}$, with $X \in \{U, H\}$

© 2025 Rao IJ et al. *JAMA Network Open*.

The model is governed by the following system of differential equations:

$$\frac{\mathrm{d}O_{2,H}}{\mathrm{d}t} = -u_{O_2}O_{2,H} + 12O_{1,H} - \left(b_H + a_H + m_{O_{2,H}}(t)\right)O_{2,H}$$

$$\frac{\mathrm{d}O_{2,U}}{\mathrm{d}t} = u_{O_2}O_{2,H} + 12O_{1,U} - \left(b_U + a_U + m_{O_{2,U}}(t)\right)O_{2,U}$$

$$\frac{\mathrm{d}O_{1,H}}{\mathrm{d}t} = -u_{O_1}O_{1,H} + \sum_{k=1}^{10} r_{k,H}\,A_{k,H} + q_H T_H + f_H I_H - \left(12 + m_{O_{1,H}}(t)\right)O_{1,H}$$

$$\frac{\mathrm{d}O_{1,U}}{\mathrm{d}t} = u_{O_1}O_{1,H} + \sum_{k=1}^{10} r_{k,U}\,A_{k,U} + q_U T_U + f_U I_U - \left(12 + m_{O_{1,U}}(t)\right)O_{1,U}$$

$$\frac{\mathrm{d}I_H}{\mathrm{d}t} = -u_I I_H + b_H O_{2,H} - \left(12 + f_H + m_{I_H}(t)\right)I_H$$

$$\frac{\mathrm{d}I_U}{\mathrm{d}t} = u_I I_H + b_U O_{2,U} - \left(12 + f_U + m_{I_U}(t)\right)I_U$$

$$\frac{\mathrm{d}T_H}{\mathrm{d}t} = -u_T T_H + 12I_H - \left(q_H + s_H + m_{T_H}(t)\right)T_H$$

$$\frac{\mathrm{d}T_U}{\mathrm{d}t} = u_T T_H + 12I_U - \left(q_U + s_U + m_{T_U}(t)\right)T_U$$

$$\frac{\mathrm{d}A_{1,H}}{\mathrm{d}t} = -u_{A_1}A_{1,H} + s_H T_H + a_H O_{2,H} - \left(1 + r_{1,H} + m_{A_{1,H}}(t)\right)A_{1,H}$$

$$\frac{\mathrm{d}A_{1,U}}{\mathrm{d}t} = u_{A_1}A_{1,H} + s_U T_U + a_U O_{2,U} - \left(1 + r_{1,U} + m_{A_{1,U}}(t)\right)A_{1,U}$$

$$\frac{\mathrm{d}A_{k,H}}{\mathrm{d}t} = -u_{A_k}A_{k,H} + A_{k-1,H} - \left(1 + r_{k,H} + m_{A_{k,H}}(t)\right)A_{k,H} \qquad k = 2, \ldots, 9$$

$$\frac{\mathrm{d}A_{k,U}}{\mathrm{d}t} = u_{A_k}A_{k,H} + A_{k-1,U} - \left(1 + r_{k,U} + m_{A_{k,U}}(t)\right)A_{k,U} \qquad k = 2, \ldots, 9$$

$$\frac{\mathrm{d}A_{10,H}}{\mathrm{d}t} = -u_{A_{10}}A_{10,H} + A_{9,H} - \left(r_{10,H} + m_{A_{10,H}}(t)\right)A_{10,H}$$

$$\frac{\mathrm{d}A_{10,U}}{\mathrm{d}t} = u_{A_{10}}A_{10,H} + A_{9,U} - \left(r_{10,U} + m_{A_{10,U}}(t)\right)A_{10,U}$$

$$\frac{\mathrm{d}D}{\mathrm{d}t} = m_{O_{2,H}}(t)O_{2,H} + m_{O_{2,U}}(t)O_{2,U} + m_{O_{1,H}}(t)O_{1,H} + m_{O_{1,U}}(t)O_{1,U} + m_{I_H}(t)I_H$$
$$+ m_{I_U}(t)I_U + m_{T_H}(t)T_H + m_{T_U}(t)T_U + \sum_{k=1}^{10}\left[m_{A_{k,H}}(t)A_{k,H} + m_{A_{k,U}}(t)A_{k,U}\right]$$

© 2025 Rao IJ et al. *JAMA Network Open*.

Model Dynamics

Figure 1 provides a schematic of the compartmental model. The boxes represent the different model compartments, and the arrows denote potential transitions between the compartments. Compartmental models are frequently used in analyses of health interventions when it is desired to track population flows between different health states, and have been used, for example, to model OUD and treatment [1, 2, 3, 4].

In Figure 1, the upper row of compartments represents housed individuals, and the lower row represents unhoused individuals. The compartments across each row represent different states of opioid use and treatment for opioid use disorder (OUD). We distinguish the first month of treatment from subsequent months. Reading from left to right in the figure, these states are as follows: out of treatment for more than 1 month; out of treatment for less than 1 month; being inducted into treatment (the first month of treatment); on treatment (after the month of induction); and then out of treatment and abstinent in the first year, second year, etc., up to 10 or more years.

We now describe the transitions between the compartments. We consider a cohort of persons experiencing homelessness (PEH) with OUD who are currently not receiving OUD treatment. In the status quo, all individuals begin in the compartment Unhoused, Out of Treatment, Month > 1 (meaning that they were not in OUD treatment during the past month). This is indicated by the lower left box in Figure 1. We consider a housing intervention where all individuals in the cohort are provided with stable housing. In this case, all individuals start in the compartment Housed, Out of Treatment, Month > 1. This is indicated by the upper left box in Figure 1. Individuals in any housed compartment (top row of Figure 1) can leave housing and move to the equivalent unhoused compartment (bottom row of Figure 1). We assume that, once unhoused, an individual does not enter housing again over the simulated time horizon but may enter and leave OUD treatment.

We model the flow of individuals into and out of OUD treatment in a manner similar to previous studies of OUD treatment [1, 4, 3]. Individuals not on treatment can enter treatment at any time. Individuals beginning treatment enter the Induction Into Treatment state; this is the first month on treatment. After the first month, if an individual remains on treatment, they enter the On Treatment state. We assume that treatment effectiveness remains constant over time for patients who continue treatment beyond the first month. Individuals on treatment can leave treatment at any time; some become abstinent (and move to the Out of Treatment and Abstinent, Year 1 compartment) and others return to untreated opioid use (and move to the Out of Treatment, Month 1 compartment). For individuals who leave treatment and become abstinent, we track the duration of abstinence, distinguishing between those in their first year of abstinence, second year, and so on, up to 10 or more years. Abstinent individuals can relapse and re-enter treatment. We assume that the likelihood of relapse decreases as the duration of abstinence increases. Death can occur from any state.

© 2025 Rao IJ et al. *JAMA Network Open*.

Parameter Value Estimation

We performed a comprehensive literature review to obtain values for model parameters. We searched PubMed using terms such as "opioid use disorder," "homelessness," "un- housed," "persons experiencing homelessness," "housing," "Housing First," "treatment," "methadone," "buprenorphine," "overdose," and "social determinants of health," alone and in combination. We also searched online for published governmental and other agency re- ports regarding homelessness, health, and opioid use disorder. We searched for all studies published up to August 2024. With the exception of one study from Scotland of mortality among persons experiencing homelessness [5], all studies used to obtain parameter esti- mates were from the US or Canada, which in many ways have similar opioid epidemics and patterns of homelessness (e.g., higher prevalence in men, strong links to substance use and mental illness) [6].

Housing intervention

We estimated values for parameters related to housing intervention effectiveness and cost from studies that reported results for Housing First programs. Such programs all offer permanent supportive housing without a requirement for sobriety or receipt of treatment, but individual programs may differ in the type and level of support services offered [7, 8]. To reflect this variability (as well as other sources of variability such as geographic region, clients served, and housing type), we performed extensive sensitivity analyses.

The length of time that clients spend in housing after being provided with permanent supportive housing varies between programs. A number of studies have examined retention in Housing First programs; none of the examined programs focused specifically on PEH with OUD. In a Housing First program for homeless Veterans, the retention rate for 107 clients receiving housing was 98% at one year (101 housed, 1 unhoused, 6 deaths) [9]. Studies of Housing First programs in several cities have found a housing retention rate of 85% after five years [8]. A study of 170 individuals in a Housing First program in Vermont found a retention rate of 85% after three years [10]. A study of several Housing First programs for individuals with serious mental illness found that of the 80 participants tracked over 12 months, 84% remained housed one year after program entry [11]. Among 111 chronically homeless people with severe alcohol problems who lived in a single-site Housing First program in Seattle, Washington, 77% remained housed at the two-year follow-up [12]. A randomized controlled trial of 423 chronically homeless individuals in Santa Clara County, California found that individuals provided with permanent supportive housing through a Housing First program remained housed for an average of 28.8 months over a four-year time horizon [13]. Based on these studies, we estimated that the average annual rate of becoming unhoused among individuals provided with housing would be 0.082. We assumed that this rate was the same for all health states (Out of Treatment, On Treatment, Out of Treatment and Abstinent, etc.). In sensitivity analysis, we considered a rate that was twice as high. We also considered the case where the rate of becoming unhoused is higher for

© 2025 Rao IJ et al. *JAMA Network Open*.

non-abstinent individuals.

The cost of stable housing provision can vary widely between programs and locales. A recent review of Housing First costs estimated a mean annual cost per person of $17,069 (Interquartile interval [IQI]: 4947 – 27,336) in 2019 USD, or $20,968 (IQI: 6075 - 33,568) in 2024 USD [14]. A study of a Housing First program in Seattle, Washington estimated an annual cost of $20,903 per person housed (expressed in 2024 USD) [15]. In the base case we estimated that the annual cost of housing would be $20,900 per person housed. We varied this value widely in sensitivity analysis.

© 2025 Rao IJ et al. *JAMA Network Open*.

OUD treatment

Housed individuals For housed individuals, rates of transition between OUD and treatment states were estimated to be the same as in several recent studies of OUD treatment [1, 4, 3]. Specifically, rates of entering OUD treatment, becoming abstinent and leaving treatment, and relapse from abstinence were drawn from a study estimating OUD state transitions [16]; the rate of leaving OUD treatment and returning to untreated opioid use was estimated from several studies of OUD treatment retention [17, 18, 19]; and the rate at which untreated individuals become abstinent without treatment was taken from a previously validated model of OUD treatment [1].

Unhoused individuals A range of studies in different settings have found that PEH with OUD have lower odds of entering OUD treatment than housed individuals with OUD [20]. For example, a study of 491 opiate-using injection drug users in Denver, Colorado found that housed individuals had higher rates of treatment entry than unhoused individuals (Odds ratio [OR] = 1.67, $p < 0.05$) [21]. A cohort study found that PEH had significantly lower odds than housed individuals of obtaining access to methadone maintenance treatment in both Oregon (OR = 0.29, $p < 0.01$) and Washington (OR = 0.55, $p < 0.01$) [22]. Retrospective analysis of 1998 data from the US Treatment Episode Dataset (TEDS) found that among both heroin users and non-users with OUD, respectively, those who reported dependent living (Adjusted Odds Ratio [AOR] = 1.90, 95% CI [1.70, 2.13]; OR = 2.23, 95% CI [1.50, 3.31]) and independent living (AOR = 2.11, 95% CI [1.90, 2.34]; OR = 2.73, 95% CI [1.88, 3.98]) had higher odds of being planned for methadone maintenance treatment than those who were homeless [23]. A study of 2006-2015 TEDS data found that PEH in detoxification had lower odds of receiving planned opiate agonist therapy than housed individuals (AOR = 0.53, $p < 0.001$) [24]. A retrospective study of 48,274 adults admitted to OUD treatment programs in Maryland found that PEH had significantly lower odds of receiving methadone or buprenorphine treatment than housed individuals (AOR = 0.46, 95% CI [0.43, 0.50]) [25]. A study of 5209 out-of-treatment drug users in five US cities found that housed individuals had higher odds of having participated in methadone detoxification (OR = 1.55, p = 0.006) and methadone maintenance (OR = 1.86, p = 0.000) than those who reported being unhoused [26]. Among 100 patients seeking office-based buprenorphine treatment at an urban medical center, recent homelessness was associated with decreased odds of beginning treatment (AOR = 0.32, 95% CI [0.10, 1.02]) [27]. Based on these studies, we estimated a hazard ratio of 0.50 for PEH entering OUD treatment compared to housed individuals.

A retrospective study of 1467 homeless or unstably housed adults receiving care at an office-based buprenorphine clinic found that retention in the treatment program was 45.2%

© 2025 Rao IJ et al. *JAMA Network Open*.

at 1 month, 21.7% at 6 months, and 11.3% at 12 months [28]. Based on this, we estimated a treatment discontinuation rate for buprenorphine among PEH of 2.18, yielding a hazard ratio of 1.35 when compared to housed individuals. This hazard ratio was also applied to treatment discontinuation for methadone.

The same study reported that continuous abstinence from illicit opioids was 28.3% at 1 month, 6.1% at 6 months, and 2.9% at 12 months [28]. Based on this, we estimated a rate of becoming abstinent and leaving OUD treatment for PEH of 0.029, resulting in a hazard ratio of 0.093 when compared to housed individuals. For the rate at which PEH not in treatment become abstinent we used the same hazard ratio as for the housed population (0.25).

Finally, we estimated the rate of relapse from abstinence among PEH. A prospective cohort study of 246 street-involved youth in Vancouver, Canada who reported abstaining from illicit drugs (opioids and/or illegal stimulants) for 6 or more months found that they were significantly more likely to report relapse than non-homeless youth (AHR = 1.43) [29]. A study of 470 patients entering short-term detoxification at an inner city inpatient unit found that unhoused individuals not using stabilization programs experienced an increased risk of return to substance use after detoxification (hazard ratio = 1.26, 95% CI [0.88, 1.80]) compared to housed individuals [30]. Thus, for the rate of relapse from abstinence we used a hazard ratio of 1.35 for unhoused versus housed individuals.

Deaths and overdose

Housed individuals Nonoverdose excess mortality for housed individuals, either in OUD treatment or not on treatment, was estimated from a systematic review of mortality among opioid users [31]. That review included studies published between 1995 and 2017 of mortality among individuals receiving medication-assistant treatment for OUD. Rates of overdose for housed individuals, in treatment or not in treatment, were estimated from previous studies [1, 3, 32, 2], as was the chance of surviving an overdose [3, 2, 33, 34]. The overdose mortality rate was calculated by multiplying the all-cause mortality rate by the percent of deaths due to overdose, and then adjusted to account for the impact of naloxone by considering the probability that naloxone is/is not administered and the probability of overdose survival with/without naloxone. The nonoverdose excess mortality rate was calculated by subtracting the overdose and background mortality rates from the all-cause mortality rate. *Unhoused individuals* A retrospective 5-year cohort study of 6757 PEH and 13,514 housed individuals in Glasgow, Scotland found that homelessness was associated with an all-cause mortality hazard ratio of 1.6 [5]. To account for this increased mortality risk, we incorporated an excess mortality rate due to homelessness for unhoused individuals. This rate was adjusted so that the all-cause mortality rate for unhoused individuals was, on average, 1.6 times higher than for housed individuals.

Similarly to housed individuals, we adjusted the overdose mortality rates for unhoused

© 2025 Rao IJ et al. *JAMA Network Open*.

individuals to take into account naloxone. We assumed that unhoused individuals have the same overdose rate as housed individuals. However, their overdose mortality rate is slightly lower as some evidence suggests that PEH have a higher chance of surviving an overdose because they are more likely to be in the presence of a bystander. A study of 6644 individuals who died from an accidental opioid overdose in Ontario, Canada found that PEH were more often in the presence of a bystander during the acute toxicity event that led to death (55.8% vs. 49.7%); and where another individual was present, more often had a resuscitation attempted (61.7% vs. 55.1%) or naloxone administered (41.2% vs. 28.9%) [34]. We followed the same methodology as for housed individuals: we used the same probability of survival with and without naloxone as for housed individuals, but estimated that the probability of an overdose being witnessed with naloxone administered is 23.0% for unhoused individuals compared to 14.4% for housed individuals.

Costs and QALY multipliers

Every health state (compartment) in the model has an associated quality-of-life multiplier and cost (Table 1). All costs are expressed in 2024 USD.

Background healthcare costs varied by age and were drawn from previous studies [35, 36]. Excess healthcare costs associated with OUD for individuals not in treatment were drawn from a study of Veterans Health Administration pharmacy and claims data from 2006-2010 [37]. Excess healthcare costs associated with OUD for individuals in OUD treatment were drawn from a retrospective claims database analysis of 13,316 patients in a large US health plan [38]. These costs were assumed to be the same for housed and unhoused individuals.

A recent systematic review of 20 Housing First Programs in the US and Canada (17 in the US and 3 in Canada; studies published from 2002 to 2019) found that the median annual healthcare cost averted by Housing First programs was $11,248 in 2019 USD [14]. Updating this value to 2024 USD, we estimated that the excess annual healthcare cost associated with homelessness is $13,823. In sensitivity analysis, we considered the conservative case of no excess healthcare cost associated with homelessness.

The annual costs of methadone and buprenorphine treatment were estimated to be $8584 and $7836, respectively [39], and the cost of naloxone per initial provision or refill was assumed to be $87 [1]. Healthcare costs per overdose include costs of calling emergency medical services [33], hospital transport [33], an emergency department visit [40], and naloxone refills [1]. More details of the cost estimation can be found in [1].

Following standard practice, we used quality-of-life multipliers for health states [41]. These multipliers are intended to represent the relative quality of life in a health state, ranging from a value of 1 for perfect health to 0 for death. Quality-of-life multipliers for health states for housed individuals were estimated from a previous model-based analysis [1], from a study of health-related quality of life by age and gender [42], and from two studies of quality-of-life improvement for individuals in OUD treatment [43, 44]. A utility multiplier

© 2025 Rao IJ et al. *JAMA Network Open*.

of 0.43 for homelessness was taken from a cross-sectional national survey conducted of 6607 middle- and low-income adults in the US [45]. This multiplier was applied to the quality adjustments for housed individuals (and ranges) to obtain estimates of quality-of-life multipliers (and ranges) for health states of PEH. We varied this value in sensitivity analysis.

Parameter probability distributions

We assumed the same form of probability distribution for each housed parameter as in a previous study [1], and we assumed the same form of probability distribution for the analogous parameter for unhoused individuals. In cases where we found multiple estimates for a given parameter value for the unhoused population, we typically used the mean value to estimate the hazard ratio associated with being unhoused, and then we created a range by applying that hazard ratio to the range for the analogous parameter for housed individuals. Parameter values were sampled according to the distributions outlined in eTable 1 and subsequently transformed to generate the rate values used in the model's differential equations.

Model Outcomes

We followed standard guidelines for cost-effectiveness analysis and measured lifetime costs and QALYs associated with the housing intervention [41]. We also calculated outcomes (overdose events and deaths) over an intermediate time period of 5 years, similar to previous analyses that focused on OUD treatment [1, 4, 3]. Costs and QALYs were discounted to the present at 3% [41].

Model Instantiation

To calculate model outcomes, we simulated cohorts of different ages, ranging from 18 to 100. We assumed that, at the start of the model time horizon, all individuals in the cohort have OUD. Our analyses make no assumption about whether individuals with OUD also use other illicit substances such as methamphetamines; we assumed the housing intervention would be offered to all individuals with OUD, regardless of whether they use other illicit substances.

For any given sex and cohort age, we simulated the cohort from that age until age 100, at which point all individuals in the cohort will have died. For each simulation we selected a value for each parameter from its associated probabiity distribution (eTable 1). We repeated the simulation 25,000 times for each parameter and age combination, with parameter values drawn each time from the probability distributions. Our model is thus inherently stochastic, reflecting uncertainty in the parameter values.

We simulated male and female cohorts of different ages, calculating health and economic outcomes for each cohort. We then obtained mean outcomes for the population, and 95% confidence intervals on those outcomes, by weighting the outcome of each age-sex combination according to the prevalence of the age-sex combination in the population with OUD

[46, 47, 48]. Further details of this simulation approach are provided elsewhere [1].

© 2025 Rao IJ et al. *JAMA Network Open*.

eTable 1. Parameter Distributions

Parameter Name	Distribution	Mean Value [95% Credible Interval]
**Transitions**		
Deaths and Overdose		
All-cause mortality on treatment	Gamma(206, 4.45×10^{-5})	0.00915 [0.00794, 0.01044]
Hazard rate ratio for all-cause mortality out of treatment compared to on treatment	Lognormal(0.850, 0.0706)	2.35 [2.04, 2.69]
Hazard rate ratio for mortality unhoused compared to housed	Uniform(1.3, 1.9)	1.6 [1.315,1.885]
Proportion of all-cause mortality due to fatal overdose	Beta(148, 171)	0.46 [0.41, 0.52]
Proportion of non-overdose excess mortality, QALY decrement, and excess healthcare cost at year 10+ of abstinence	Uniform(0.01, 0.11)	0.06 [0.0125, 0.1075]
Overdose Survival Probability		
Probability of surviving an overdose without naloxone, probability of surviving an overdose with naloxone	Bivariate Beta(54.67, 0.044, 1.29, 5.57)[1]	0.899 [0.799, 0.954]<br>0.909 [0.826, 0.967]
Fentanyl hazards		
Fentanyl penetration in drug supply	Gamma(384.16, 0.001)	0.44 [0.397, 0.485]
Overdose risk multiplier	Gamma(100, 0.02)	2.0 [1.627, 2.411]
Mortality multiplier	Gamma(96.04, 0.02)	2.0 [1.620, 2.419]

[1] Description of this type of distribution can be found in Olkin I, Trikalinos TA**.** Constructions for a bivariate beta distribution. Stat Prob Ltrs. 2015;96(1):54-60.

© 2025 Rao IJ et al. *JAMA Network Open*.

Parameter Name	Distribution	Mean Value [95% Credible Interval]
Treatment Discontinuation		
Hazard rate ratio for the treatment discontinuation rate for buprenorphine and methadone[2]	Multivariate Lognormal $m = 0.00295 \quad -0.436$ $\Sigma = \begin{bmatrix} 0.0201 & 0.0201 \\ 0.0201 & 0.0492 \end{bmatrix}$	1.01 [0.76, 1.32] 0.66 [0.42, 0.99]
Other Transitions		
Rate of becoming unhoused	Gamma(384, 0.000213)	0.0820 [0.0739, 0.0903]
Rate of reentry to treatment (housed, from out of treatment > 1 month)	Gamma(185.90, 0.0023)	0.426 [0.367, 0.489]
Rate of reentry to treatment (unhoused, from out of treatment > 1 month) for unhoused individuals	Gamma(185.90, 0.0011)	0.213 [0.184, 0.245]
Rate of becoming abstinent from treatment (housed)	Gamma(885.96, 0.000357)	0.316 [0.296, 0.337]
Rate of becoming abstinent from treatment (unhoused)	Gamma(885.96, 0.000033)	0.029 [0.028, 0.031]
Rate of relapse from abstinence < 1 year (housed)	Gamma(227.50, 0.00167)	0.379 [0.331, 0.430]
Rate of relapse from abstinence < 1 year (unhoused)	Gamma(227.50, 0.00224)	0.510 [0.445, 0.578]
Hazard rate ratio for becoming abstinent while out of treatment compared to in treatment	Uniform(0, 0.5)	0.25 [0.0125, 0.4875]
**Annual Costs**		

[2] The treatment discontinuation rate was calculated from a previous study of opioid treatment (Fairley M, Humphreys K, Joyce VR, et al. Cost-effectiveness of Treatments for Opioid Use Disorder. JAMA Psychiatry. 2021;78(7):767–777). It was calculated relative to naltrexone, which assumed a gamma distribution with a shape parameter $\alpha = 33.24$, and scale parameter $\theta = 0.048$ for housed individuals, and a gamma distribution with $\alpha = 33.24$, and $\theta = 0.065$ for unhoused individuals. On top of this was applied a multivariate lognormal distribution as shown in the table.

© 2025 Rao IJ et al. *JAMA Network Open.*

**Parameter Name**	**Distribution**	**Mean Value [95% Credible Interval]**
Excess cost for OUD out of treatment	Gamma(384.1, 23.0)[3]	8,826 [7,965, 9,731]
Multiplier for excess cost for OUD in treatment	Uniform(0.4, 1.2)	0.8 [0.42, 1.18]
Excess healthcare cost for homelessness	Gamma(384, 35.98)	13,823 [12,475, 15,257]
Methadone cost	Gamma(384, 22.35)	8,584 [7,747, 9,464]
Buprenorphine cost	Gamma(384, 20.40)	7,835 [7,071, 8,638]
Cost of overdose without naloxone, cost of overdose with naloxone, and cost of naloxone refill	Multivariate Lognormal $m = [7.99 \quad 7.76 \quad 4.47]$ $\Sigma = \begin{bmatrix} 0.1526 & 0.1533 & 2.759´10^{-5} \\ 0.1533 & 0.1547 & 4.541´10^{-5} \\ 2.759´10^{-5} & ´10^{-5} & 0.0026 \end{bmatrix}$	3,173 [1,367, 6343]<br>2,538 [1,085, 5,101]<br>87 [79 , 96]
**Quality-of-Life Multipliers**		
QALY weight for on treatment (housed)	Beta(1,263, 489)	0.721 [0.700, 0.742]
QALY weight for on treatment (unhoused)	Beta(3,111, 6,831)	0.313 [0.304, 0.322]
QALY weight for out of treatment (housed)	Beta(3,642, 1,814)	0.668 [0.655, 0.680]
QALY weight for out of treatment (unhoused)	Beta(7,780, 19,077)	0.290 [0.284, 0.295]

Abbreviations: IDU = injection drug use, OUD = opioid use disorder, QALY = quality-adjusted life year

[3] We use the convention Gamma(α,θ) where α is the shape parameter and θ is the scale parameter.

© 2025 Rao IJ et al. *JAMA Network Open*.

eTable 2: Sensitivity Analysis: Outcomes Assuming OUD Treatment with Methadone (Mean Values and 95% Confidence Intervals)

	Over 5 years, per 1,000 individuals				Lifetime, per person discounted				
**Scenario**	**Fatal overdoses**	**Nonfatal overdoses**	**Total overdoses**	**Deaths**	**QALYs**	**Health care costs**[1]	**Housing costs**[1]	**Total costs**[1]	**ICER**[1]
**Higher QALYs for PEH**[2]									
Status quo	58<br>[44-77]	495<br>[221-1011]	553<br>[275-1076]	190<br>[152-237]	5.13<br>[4.49-5.72]	449<br>[388-510]	0<br>[0-0]	449<br>[388-510]	
Housing intervention	53<br>[39-76]	440<br>[192-928]	493<br>[237-986]	140<br>[114-184]	8.13<br>[7.28-8.83]	397<br>[346-442]	148<br>[138-157]	545<br>[486-594]	32.0<br>[25.5-38.1]
**Excess healthcare cost associated with being unhoused is decreased by 50%**									
Status quo	59<br>[44-78]	496<br>[220-1015]	554<br>[274-1080]	191<br>[153-237]	3.71<br>[3.26-4.14]	362<br>[313-410]	0<br>[0-0]	362<br>[313-410]	
Housing intervention: Cost of housing is as in the base case	53<br>[39-76]	440<br>[191-927]	493<br>[237-986]	139<br>[115-184]	7.30<br>[6.59-7.87]	348<br>[304-386]	148<br>[138-157]	496<br>[444-539]	37.6<br>[32.9-42.2]
Housing intervention: Cost of housing is increased by 50%	53<br>[39-76]	443<br>[191-923]	496<br>[238-980]	140<br>[115-183]	7.30<br>[6.57-7.87]	348<br>[305-386]	223<br>[207-235]	570<br>[515-615]	58.3<br>[53.2-63.7]
Housing intervention: Cost of housing is doubled	53<br>[39-76]	445<br>[193-947]	498<br>[239-1009]	140<br>[115-185]	7.30<br>[6.56-7.86]	348<br>[305-386]	297<br>[275-313]	644<br>[582-692]	79.0<br>[72.9-85.4]
**No excess healthcare cost associated with being unhoused**									
Status quo	58<br>[44-77]	497<br>[221-1022]	555<br>[274-1085]	190<br>[152-236]	3.72<br>[3.26-4.14]	275<br>[237-312]	0<br>[0-0]	275<br>[237-312]	

© 2025 Rao IJ et al. *JAMA Network Open.*

	Over 5 years, per 1,000 individuals				Lifetime, per person discounted				
**Scenario**	**Fatal overdoses**	**Nonfatal overdoses**	**Total overdoses**	**Deaths**	**QALYs**	**Health care costs**[1]	**Housing costs**[1]	**Total costs**[1]	**ICER**[1]
Housing intervention: Cost of housing is as in the base case	53 [39-75]	442 [192-917]	495 [238-981]	139 [114-182]	7.30 [6.61-7.88]	299 [264-332]	148 [138-157]	447 [404-484]	48.3 [43.6-53.0]
Housing intervention: Cost of housing is increased by 50%	53 [39-75]	442 [189-924]	495 [235-982]	139 [114-184]	7.30 [6.57-7.88]	299 [263-332]	222 [206-235]	522 [472-562]	69.0 [63.2-74.9]
Housing intervention: Cost of housing is doubled	53 [39-76]	441 [192-921]	494 [239-983]	140 [114-185]	7.30 [6.57-7.88]	299 [262-332]	297 [275-313]	596 [541-638]	89.8 [82.8-97.4]

[1]All costs expressed in 1,000 $2024; ICER = incremental cost-effectiveness ratio, calculated as cost per QALY gained compared to the status quo
[2]Baseline quality-of-life adjustment associated with being unhoused is increased from 0.43 to 0.6.

© 2025 Rao IJ et al. *JAMA Network Open*.

eTable 3: Outcomes for Each Scenario, Assuming OUD Treatment with Buprenorphine (Mean Values and 95% Confidence Intervals)

	Over 5 years, per 1,000 individuals				Lifetime, per person discounted				
**Scenario**	**Fatal overdoses**	**Nonfatal overdoses**	**Total overdoses**	**Deaths**	**QALYs**	**Health care costs[1]**	**Housing costs[1]**	**Total costs[1]**	**ICER[1]**
Status quo	56 [43-71]	477 [213-967]	533 [264-1026]	186 [150-227]	3.74 [3.33-4.14]	449 [394-505]	0 [0-0]	449 [394-505]	
**Base case analysis**									
Housing intervention	50 [38-64]	414 [183-843]	464 [228-898]	132 [113-154]	7.33 [6.78-7.86]	399 [361-437]	150 [142-157]	549 [507-590]	27.6 [22.3-32.9]
**Sensitivity analyses**									
Lower treatment success for housed individuals[2]	53 [41-68]	441 [195-890]	494 [244-949]	137 [117-159]	7.08 [6.57-7.57]	395 [358-434]	148 [140-156]	543 [502-585]	28.0 [22.5-33.2]
Housing not associated with changes in OUD treatment entry or outcomes[3]	59 [45-75]	489 [217-1003]	548 [272-1066]	146 [125-170]	6.66 [6.24-7.08]	389 [349-429]	145 [136-153]	534 [490-577]	28.8 [23.0-34.0]
Lower probability of witnessing an overdose for housed individuals[4]	51 [39-66]	416 [185-849]	467 [231-905]	133 [114-155]	7.32 [6.77-7.83]	398 [360-436]	150 [142-157]	547 [506-588]	27.4 [22.0-32.6]
Higher rate of becoming unhoused[5]	51 [39-65]	425 [186-856]	475 [232-910]	140 [119-165]	6.00 [5.55-6.42]	419 [375-463]	95 [90-100]	514 [468-558]	28.5 [23.0-33.7]
Higher rate of becoming unhoused for nonabstinent individuals[6]	50 [38-65]	416 [183-841]	466 [230-894]	136 [116-159]	6.74 [6.18-7.27]	407 [366-450]	125 [117-133]	532 [488-577]	27.7 [22.3-33.0]

© 2025 Rao IJ et al. *JAMA Network Open.*

	Over 5 years, per 1,000 individuals				Lifetime, per person discounted				
**Scenario**	**Fatal overdoses**	**Nonfatal overdoses**	**Total overdoses**	**Deaths**	**QALYs**	**Health care costs[1]**	**Housing costs[1]**	**Total costs[1]**	**ICER[1]**
Housing not associated with changes in excess mortality due to homelessness[7]	49<br>[37-63]	404<br>[181-833]	452<br>[225-886]	168<br>[136-206]	6.85<br>[6.18-7.50]	373<br>[327-418]	140<br>[131-149]	513<br>[459-564]	20.4<br>[16.5-24.2]
Doubled cost of housing	50<br>[38-65]	414<br>[188-831]	464<br>[233-886]	132<br>[112-154]	7.33<br>[6.77-7.86]	399<br>[361-438]	300<br>[285-314]	699<br>[652-744]	69.5<br>[63.3-75.9]

[1]All costs expressed in 1,000 $2024; ICER = incremental cost-effectiveness ratio, calculated as cost per QALY gained compared to the status quo

[2]Rate of entry into treatment and rate of becoming abstinent is 20% lower, rate of treatment discontinuation and rate of relapse from abstinence is 20% higher.

[3]Rates of entry into treatment and treatment outcomes for housed individuals are the same as for unhoused individuals.

[4]Probability of witnessing an overdose for housed individuals is 80% lower than for housed individuals.

[5]Rate of becoming unhoused is twice as high.

[6]The rate of becoming unhoused is 50% higher for individuals out of treatment, 25% higher for those in treatment, and unchanged for abstinent individuals.

[7]Excess mortality due to homelessness for housed individuals is the same as for unhoused individuals.

© 2025 Rao IJ et al. *JAMA Network Open*.

eTable 4: Sensitivity Analysis: Outcomes Assuming OUD Treatment with Buprenorphine (Mean Values and 95% Confidence Intervals)

	Over 5 years, per 1,000 individuals				Lifetime, per person discounted				
**Scenario**	**Fatal overdoses**	**Nonfatal overdoses**	**Total overdoses**	**Deaths**	**QALYs**	**Health care costs**[1]	**Housing costs**[1]	**Total costs**[1]	**ICER**[1]
**Higher QALYs for PEH**[2]									
Status quo	56 [43-71]	476 [216-968]	532 [268-1029]	186 [150-227]	5.16 [4.59-5.72]	449 [394-506]	0 [0-0]	449 [394-506]	
Housing intervention	50 [38-64]	413 [186-843]	463 [231-897]	132 [112-154]	8.17 [7.52-8.79]	399 [359-438]	150 [142-157]	549 [506-591]	33.0 [26.8-39.0]
**Excess healthcare cost associated with being unhoused is decreased by 50%**									
Status quo	56 [43-71]	478 [215-966]	534 [268-1021]	186 [149-226]	3.74 [3.33-4.14]	361 [317-406]	0 [0-0]	361 [317-406]	
Housing intervention: Cost of housing is as in the base case	50 [38-64]	416 [186-850]	465 [231-903]	132 [113-154]	7.33 [6.78-7.85]	349 [317-382]	150 [142-157]	499 [463-535]	38.4 [33.7-43.0]
Housing intervention: Cost of housing is increased by 50%	50 [38-64]	413 [184-839]	463 [228-895]	132 [113-154]	7.34 [6.78-7.86]	349 [317-382]	225 [214-236]	574 [536-612]	59.2 [53.8-64.7]
Housing intervention: Cost of housing is doubled	50 [38-64]	412 [185-836]	462 [230-889]	132 [113-154]	7.33 [6.78-7.84]	349 [317-383]	300 [285-314]	649 [609-689]	80.2 [73.7-87.0]
**No excess healthcare cost associated with being unhoused**									
Status quo	56 [43-72]	480 [217-973]	536 [268-1035]	186 [150-227]	3.74 [3.33-4.15]	274 [239-309]	0 [0-0]	274 [239-309]	

© 2025 Rao IJ et al. *JAMA Network Open.*

	Over 5 years, per 1,000 individuals				Lifetime, per person discounted				
**Scenario**	**Fatal overdoses**	**Nonfatal overdoses**	**Total overdoses**	**Deaths**	**QALYs**	**Health care costs**[1]	**Housing costs**[1]	**Total costs**[1]	**ICER**[1]
Housing intervention: Cost of housing is as in the base case	50 [38-65]	417 [186-858]	467 [232-912]	132 [113-154]	7.33 [6.78-7.85]	300 [272-328]	150 [142-157]	449 [418-481]	49.1 [44.2-53.9]
Housing intervention: Cost of housing is increased by 50%	50 [38-65]	414 [186-853]	464 [231-906]	132 [113-154]	7.33 [6.79-7.85]	299 [272-328]	225 [214-236]	524 [491-557]	70.0 [63.9-76.3]
Housing intervention: Cost of housing is doubled	50 [38-64]	414 [187-859]	464 [232-914]	132 [113-154]	7.33 [6.78-7.85]	299 [272-327]	300 [285-314]	599 [563-634]	90.8 [83.4-98.9]

[1]All costs expressed in 1,000 $2024; ICER = incremental cost-effectiveness ratio, calculated as cost per QALY gained compared to the status quo
[2]Baseline quality-of-life adjustment associated with being unhoused is increased from 0.43 to 0.6.

© 2025 Rao IJ et al. *JAMA Network Open*.

eRESULTS

This section presents results of additional sensitivity analyses not described in the main text.

Higher discount rate If the discount rate is increased from 3% to 5%, health outcomes are unchanged but the housing intervention costs $25,100 per QALY gained compared to the status quo, reflecting a shift in the present value of future costs and benefits.

Outreach costs The base case did not include outreach costs to locate unhoused individuals for placement in housing. Several studies have estimated that 85-90% of unhoused individuals reached through outreach would accept permanent supportive housing [13, 49]. With an acceptance rate of 85%, we estimate that if outreach costs are less than $450,000, the housing intervention costs less than $150,000 per QALY gained. If outreach costs are less than $300,000, the housing intervention costs less than $100,000 per QALY gained, and if outreach costs are under $150,000, the housing intervention costs less than $50,000 per QALY gained.

eReferences

[1] M Fairley *et al.*, "Cost-effectiveness of treatments for opioid use disorder," *JAMA Psychiatry,* vol. 78, no. 7, pp. 776–77, 2021.

[2] T. Y. Lim *et al.*, "Modeling the evolution of the US opioid crisis for national policy development," *PNAS*, vol. 119, no. 23, 2022.

[3] G. Qian, K. Humphreys, J. D. Goldhaber-Fiebert, and M. L. Brandeau, "Estimated effectiveness and cost-effectiveness of opioid use disorder treatment under proposed U.S. regulatory relaxations: A model-based analysis," *Drug Alcohol Depend*, vol. 256, p. 111 112, 2024.

[4] G. Qian, I. J. Rao, K. Humphreys, D. K. Owens, and M. L. Brandeau, "Cost-effectiveness of office-based buprenorphine treatment for opioid use disorder," *Drug Alcohol Depend*, vol. 243, p. 109 762, 2023.

[5] D. S. Morrison, "Homelessness as an independent risk factor for mortality: Results from a retrospective cohort study," *Int J Epidemiol*, vol. 38, no. 3, 877–883, 2009.

[6] K. Humphreys *et al.*, "Responding to the opioid crisis in North America and beyond: Recommendations of the Stanford-Lancet Commission," *Lancet*, vol. 399, no. 10324, pp. 555–604, 2022.

[7] US Department of Housing and Urban Development, Office of Policy Development and Research, "Housing First: A Review of the Evidence," 2023. [Online]. Available: https://www.huduser.gov/portal/periodicals/em/spring-summer-23/highlight2.html.

[8] S. G. Pfefferle, S. S. Karon, and B. Wyant, "Choice matters: Housing models that may promote recovery for individuals and families facing opioid use disorder," Tech. Rep., 2019. [Online]. Available: https://aspe.hhs.gov/sites/default/files/private/pdf/261936/Choice.pdf.

[9] A. E. Montgomery, L. L. Hill, V. Kane, and D. P. Culhane, "Housing chronically homeless Veterans: Evaluating the efficacy of a Housing First approach to HUD-VASH," *J Commun Psychol*, vol. 41, no. 4, pp. 505–514, 2013.

[10] A. Stefancic, B. F. Henwood, H. Melton, S. M. Shin, R. Lawrence-Gomez, and S. Tsemberis, "Implementing Housing First in rural areas: Pathways Vermont," *Am J Public Health*, vol. 103 Suppl 2, no. Suppl 2, S206–9, 2013.

[11] C. Pearson, A. E. Montgomery, and G. Locke, "Housing stability among homeless individuals with serious mental illness participating in Housing First programs," *J Commun Psychol*, vol. 37, no. 3, pp. 404–417, 2009.

© 2025 Rao IJ et al. *JAMA Network Open.*

[12] S. E. Collins, D. K. Malone, and S. L. Clifasefi, "Housing retention in single-site housing first for chronically homeless individuals with severe alcohol problems," *Am J Public Health*, vol. 103 Suppl 2, no. Suppl 2, S269–74, 2013.

[13] M. C. Raven, M. J. Niedzwiecki, and M. Kushel, "A randomized trial of permanent supportive housing for chronically homeless persons with high use of publicly funded services," *Health Serv Res*, vol. 55, no. S2, 797–806, 2020.

[14] V. Jacob *et al.*, "Permanent supportive housing with Housing First: Findings from a community guide systematic economic review," *Am J Prev Med*, vol. 62, no. 3, e188–e201, 2022.

[15] M. E. Larimer, "Health care and public service use and costs before and after provision of housing for chronically homeless persons with severe alcohol problems," *JAMA*, vol. 301, no. 13, p. 1349, 2009.

[16] E. Krebs *et al.*, "Estimating state transitions for opioid use disorders," *Med Decis Making*, vol. 37, no. 5, pp. 483–497, 2017.

[17] Y. I. Hser *et al.*, "Treatment retention among patients randomized to buprenorphine/naloxone compared to methadone in a multi-site trial," *Addiction*, vol. 109, no. 1, pp. 79–87, 2014.

[18] A. M. Neumann *et al.*, "A preliminary study comparing methadone and buprenorphine in patients with chronic pain and coexistent opioid addiction," *J Addict Dis*, vol. 32, no. 1, pp. 68–78, 2013.

[19] J. S. Potter *et al.*, "Buprenorphine/naloxone and methadone maintenance treatment outcomes for opioid analgesic, heroin, and combined users: Findings from starting treatment with agonist replacement therapies (START)," *J Stud Alcohol Drugs*, vol. 74, no. 4, pp. 605–13, 2013.

[20] M. F. McLaughlin, R. Li, N. D. Carrero, P. A. Bain, and A. Chatterjee, "Opioid use disorder treatment for people experiencing homelessness: A scoping review," *Drug Alcohol Depend*, vol. 224, p. 108 717, 2021.

[21] K. F. Corsi, C. F. Kwiatkowski, and R. E. Booth, "Treatment entry and predictors among opiate-using injection drug users," *Am J Drug Alcohol Abuse*, vol. 33, no. 1, pp. 121–7, 2007.

[22] D. Deck and M. J. Carlson, "Access to publicly funded methadone maintenance treatment in two Western states," *J Behav Health Serv Res*, vol. 31, no. 2, pp. 164–77, 2004.

[23] P. A. Rivers, A. Dobalian, T. J. Oyana, and S. Bae, "Socioeconomic determinants of planned methadone treatment," *Am J Health Behav*, vol. 30, no. 5, pp. 451–9, 2006.

© 2025 Rao IJ et al. *JAMA Network Open.*

[24] K. E. Dunn, A. S. Huhn, and E. C. Strain, "Differential adoption of opioid agonist treatments in detoxification and outpatient settings," *J Subst Abuse Treat*, vol. 107, pp. 24–28, 2019.

[25] N. Krawczyk *et al.*, "Opioid agonist treatment and fatal overdose risk in a state-wide US population receiving opioid use disorder services," *Addiction*, vol. 115, no. 9, 1683–1694, 2020.

[26] D. Royse *et al.*, "Homelessness and gender in out-of-treatment drug users," *Am J Drug Alcohol Abuse*, vol. 26, no. 2, pp. 283–96, 2000.

[27] C. B. Simon, J. I. Tsui, J. O. Merrill, A. Adwell, E. Tamru, and J. W. Klein, "Linking patients with buprenorphine treatment in primary care: Predictors of engagement," *Drug Alcohol Depend*, vol. 181, pp. 58–62, 2017.

[28] D. R. Fine, E. Lewis, K. Weinstock, J. Wright, J. M. Gaeta, and T. P. Baggett, "Office-based addiction treatment retention and mortality among people experiencing homelessness," *JAMA Netw Open*, vol. 4, no. 3, e210477, 2021.

[29] J. Goldman-Hasbun, E. Nosova, T. Kerr, E. Wood, and K. DeBeck, "Homelessness and incarceration associated with relapse into stimulant and opioid use among youth who are street-involved in Vancouver, Canada," *Drug Alcohol Rev*, vol. 38, no. 4, 428–434, 2019.

[30] S. G. Kertesz, N. J. Horton, P. D. Friedmann, R. Saitz, and J. H. Samet, "Slowing the revolving door: Stabilization programs reduce homeless persons' substance use after detoxification," *J Subst Abuse Treat*, vol. 24, no. 3, 197–207, 2003.

[31] J. Ma *et al.*, "Effects of medication-assisted treatment on mortality among opioids users: A systematic review and meta-analysis," *Mol Psychiatry*, vol. 24, pp. 1868–83, 2019.

[32] E. Kelty, D. Joyce, and G. Hulse, "A retrospective cohort study of mortality rates in patients with an opioid use disorder treated with implant naltrexone, oral methadone or sublingual buprenorphine," *Am J Drug Alcohol Abuse*, vol. 45, no. 3, pp. 285–291, 2019.

[33] P. O. Coffin and S. D. Sullivan, "Cost-effectiveness of distributing naloxone to heroin users for lay overdose reversal," *Ann Intern Med*, vol. 158, no. 1, pp. 1–9, 2013.

[34] R. G. Booth *et al.*, "Opioid-related overdose deaths among people experiencing homelessness, 2017 to 2021: A population-based analysis using coroner and health administrative data from Ontario, Canada," *Addiction*, vol. 119, no. 2, pp. 334–344, 2024.

[35] S. Liu, L. E. Cipriano, M. Holodniy, D. K. Owens, and J. D. Goldhaber-Fiebert, "New protease inhibitors for the treatment of chronic hepatitis C: A cost-effectiveness analysis," *Ann Intern Med*, vol. 156, no. 4, pp. 279–90, 2012.

© 2025 Rao IJ et al. *JAMA Network Open*.

[36] E. Meara, C. White, and D. M. Cutler, "Trends in medical spending by age, 1963-2000," *Health Aff (Millwood)*, vol. 23, no. 4, pp. 176–83, 2004.

[37] O. Baser, L. Xie, J. Mardekian, D. Schaaf, L. Wang, and A. V. Joshi, "Prevalence of diagnosed opioid abuse and its economic burden in the Veterans Health Administration," *Pain Pract*, vol. 14, no. 5, pp. 437–45, 2014.

[38] O. Baser, M. Chalk, D. A. Fiellin, and D. R. Gastfriend, "Cost and utilization outcomes of opioid-dependence treatments," *Am J Manag Care*, vol. 17 Suppl 8, S235–48, 2011.

[39] US Department of Defense, "TRICARE; mental health and substance use disorder treatment," *Federal Register*, vol. 81, no. 171, pp. 61 068–61 098, 2016.

[40] Premier Inc., "Opioid overdoses costing U.S. hospitals an estimated $11 billion annually," 2019. [Online]. Available: https://www.premierinc.com/newsroom/press-releases/opioid-overdoses-costing-u-s-hospitals-an-estimated-11-billion-annually.

[41] G. D. Sanders *et al.*, "Recommendations for conduct, methodological practices, and reporting of cost-effectiveness analyses: Second Panel on Cost-Effectiveness in Health and Medicine," *JAMA*, vol. 316, no. 10, pp. 1093–103, 2016.

[42] D. G. Fryback *et al.*, "US norms for six generic health-related quality-of-life indexes from the National Health Measurement Study," *Med Care*, vol. 45, no. 12, pp. 1162–70, 2007.

[43] E. Krebs *et al.*, "Cost-effectiveness of publicly funded treatment of opioid use disorder in California," *Ann Intern Med*, vol. 168, no. 1, pp. 10–19, 2018.

[44] B. Nosyk *et al.*, "Short term health-related quality of life improvement during opioid agonist treatment," *Drug Alcohol Depend*, vol. 157, pp. 121–8, 2015.

[45] S. S. Rajan and J. Tsai, "Estimation of utility values for computing quality-adjusted life years associated with homelessness," *Med Care*, vol. 59, no. 12, pp. 1115–1121, 2021.

[46] M. Kushel *et al.*, "Toward a New Understanding: The California Study of People Experiencing Homelessness," USCF Benioff Homelessness and Housing Initiative, 2023. [Online]. Available: https://homelessness.ucsf.edu/our-impact/studies/california-statewide-study-people-experiencing-homelessness.

[47] Substance Abuse and Mental Health Services Administration, *National Survey on Drug Use and Health, 2018 (NSDUH-2018)*, Accessed: 2024-05-23, 2018. [Online]. Available: https://www.datafiles.samhsa.gov/studydataset/national-survey-drug-use-and-health-2018-nsduh-2018-ds0001-nid18758.

[48] U.S. Census Bureau, *American Community Survey Public Use Microdata Sample (ACS PUMS) 5-Year Data, 2018*, Accessed: 2024-05-23, 2018. [Online]. Available: https://data.census.gov/mdat/#/search?ds=ACSPUMS5Y2018&cv=SEX&rv=AGEP&wt=PWGTP.

[49] US Interagency Council on Homelessness, "Homelessness data and trends," Report, 2021. [Online]. Available: https://www.usich.gov/guidance-reports-data/data-trends.

[50] I. Olkin and T. A. Trikalinos, "Constructions for a bivariate beta distribution," *Stat Prob Ltrs*, vol. 96, no. C, pp. 54–60, 2015.

© 2025 Rao IJ et al. *JAMA Network Open*.
