# Supplementary material for: Modeling Health and Economic Outcomes of Providing Stable Housing to Homeless Adults With OUD
Source: JAMA Netw Open. 2025 Jun 27;8(6):e2517103. doi: 10.1001/jamanetworkopen.2025.17103 (PMC12205399; doi:10.1001/jamanetworkopen.2025.17103)
Supplement: Supplement 2. — Data Sharing Statement [file jamanetwopen-e2517103-s002.pdf]

# Data Sharing Statement

Rao. Modeling Health and Economic Outcomes of Providing Stable Housing to Homeless Adults With OUD. *JAMA Netw Open*. Published June 25, 2025.
doi:10.1001/jamanetworkopen.2025.17103

**Data**
**Data available:** No
